# 1-(3,5-Dimethyl­phen­yl)-2-(4-fluoro­phen­yl)-1*H*-phenanthro[9,10-*d*]imidazole

**DOI:** 10.1107/S1600536813003486

**Published:** 2013-02-09

**Authors:** R. Sathishkumar, T. Mohandas, P. Sakthivel, J. Jayabharathi

**Affiliations:** a Department of Chemistry, Annamalai University, Annamalainagar 608 002, India; b Shri Angalamman College of Engineering and Technology, Siruganoor, Tiruchirappalli 621 105, India; c Department of Physics, Urumu Dhanalakshmi College, Tiruchirappalli 620 019, India

## Abstract

In the title compound, C_29_H_21_FN_2_, the phenanthro tricyclic ring system is essentially planar with a maximum deviation of 0.030 (2) Å and makes dihedral angles between of 77.96 (6) and 37.18 (7)° with the dimethyl­phenyl and fluoro­phenyl rings, respectively. The crystal packing features weak C—H⋯π inter­actions involving the dimethyl­phenyl and other phenyl rings.

## Related literature

For the use of phenanthroline derivatives in the construction of mol­ecular devices, see: Yamada *et al.* (1992 ▶). For the biological activity of imidazole, see: Nebert & Gonzalez (1987 ▶). For related metallo-supra­molecular chemistry, see: Lehn (1996 ▶). For applications of complexes based on phenanthroline, see: Walters *et al.* (2000 ▶); Peng *et al.* (1997 ▶); Hara *et al.* (2001 ▶).
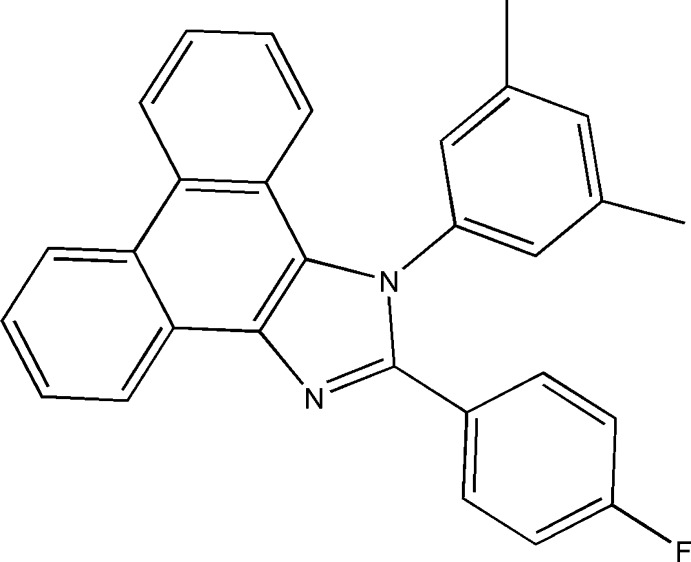


## Experimental

### 

#### Crystal data

C_29_H_21_FN_2_
*M* *_r_* = 416.48
Monoclinic, 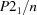

*a* = 8.5680 (2) Å
*b* = 10.6070 (3) Å
*c* = 23.6900 (6) Å
β = 93.899 (1)°
*V* = 2147.98 (10) Å^3^
*Z* = 4
Mo *K*α radiation
μ = 0.08 mm^−1^
*T* = 295 K
0.30 × 0.20 × 0.20 mm

#### Data collection

Bruker Kappa APEXII CCD diffractometer
Absorption correction: multi-scan (*SADABS*; Bruker, 2008 ▶) *T*_min_ = 0.952, *T*_max_ = 0.995
19934 measured reflections
3777 independent reflections
2957 reflections with *I* > 2σ(*I*)
*R*_int_ = 0.034

#### Refinement

*R*[*F* ^2^ > 2σ(*F* ^2^)] = 0.039
*wR*(*F* ^2^) = 0.109
*S* = 1.02
3777 reflections
292 parameters
H-atom parameters constrained
Δρ_max_ = 0.24 e Å^−3^
Δρ_min_ = −0.15 e Å^−3^

### 

Data collection: *APEX2* (Bruker, 2008 ▶); cell refinement: *APEX2* and *SAINT* (Bruker, 2008 ▶); data reduction: *SAINT*; program(s) used to solve structure: *SHELXS97* (Sheldrick, 2008 ▶); program(s) used to refine structure: *SHELXL97* (Sheldrick, 2008 ▶); molecular graphics: *ORTEP-3 for Windows* (Farrugia, 2012 ▶); software used to prepare material for publication: *PLATON* (Spek, 2009 ▶).

## Supplementary Material

Crystal structure: contains datablock(s) global, I. DOI: 10.1107/S1600536813003486/rk2393sup1.cif

Structure factors: contains datablock(s) I. DOI: 10.1107/S1600536813003486/rk2393Isup2.hkl

Additional supplementary materials:  crystallographic information; 3D view; checkCIF report

## Figures and Tables

**Table 1:** Hydrogen-bond geometry (Å, °) *Cg*1 and *Cg*2 are the centroids of the C7/C8/C13/C14/C19/C20 and C8–C13 rings, respectively.

*D*—H⋯*A*	*D*—H	H⋯*A*	*D*⋯*A*	*D*—H⋯*A*
C9—H9⋯*Cg*1	0.93	2.97	3.84	156
C6—H6⋯*Cg*2^i^	0.93	2.97	3.70	155

Symmetry code: (i) 
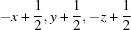
.

**Fig. 1.**
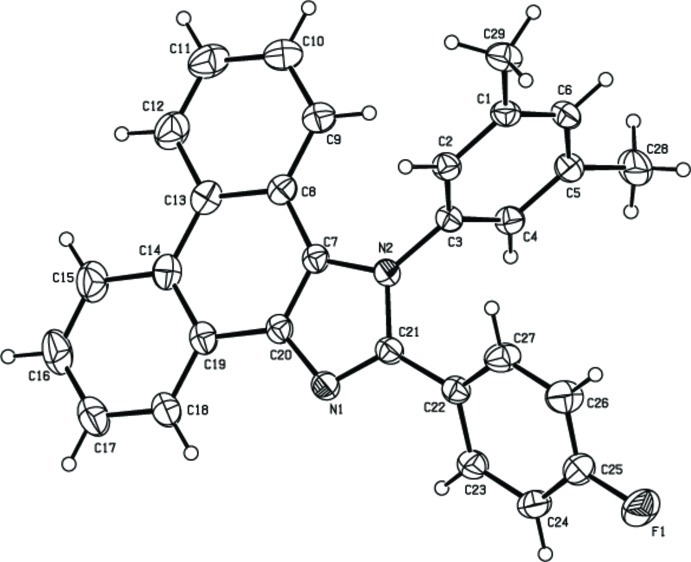
The molecular structure with the atom numbering scheme. Displacement ellipsoids are drawn at the 30% probability level. H atoms are presented as spheres with arbitrary radius.

**Fig. 2.**
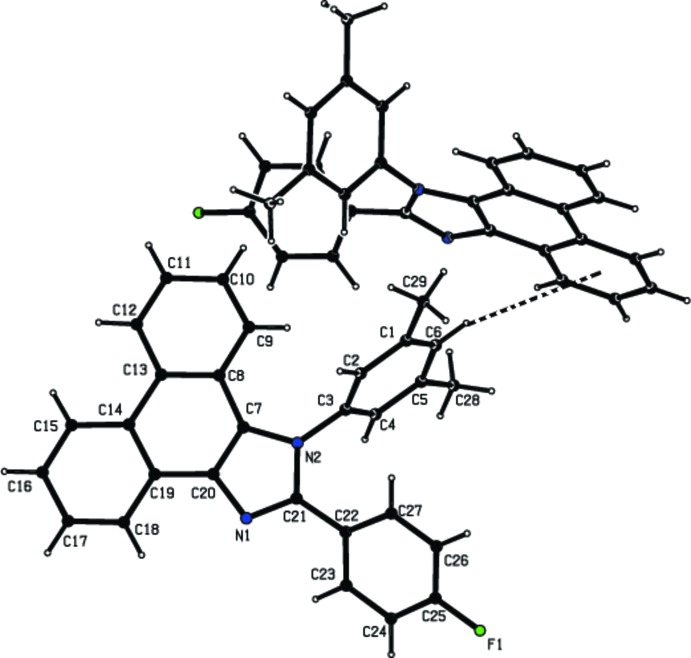
A packing diagram of the title compound. The molecules are connected by C–H···π interactions as shown by dashed line.

## supplementary crystallographic information

### Comment

The derivatives of phenanthroline, which have excellent hole blocking and electron transporting properties, are likely to have interesting value in the construction of molecular devices (Yamada *et al.*, 1992).

Indeed, various imidazole derivatives have shown a broad range of bioactivities, such as antineoplastic, immunosuppressive and anti-inflammatory activities (Nebert *et al.*, 1987).

The large variety of complexes based on phenanthroline and its derivatives allows the formation of many different molecular systems with various applications ranging from metallo-supramolecular chemistry (Lehn *et al.*, 1996), metal sensors (Walters *et al.*, 2000), molecular electronics (Peng *et al.*, 1997) and photo sensitizers (Hara *et al.*, 2001).

The molecular structure is shown in Fig.1. The phenanthro tricycle is essentialy planar. The dihedral angles between phenanthro tricycle to the dimethylphenyl is 77.96 (6)° and to that of fluorophenyl ring is 37.18 (7)° respectively.

Further the crystal is stabilized by intermolecular C–H···π interactions (Table 1), where Cg1 is the centre of gravity of C7/C8/C13/C14/C19/C20 and Cg2 is the centre of gravity of (C8-C13). The symmetry code are: (i) 1/2-*x*, 1/2+*y*, 1/2-*z*.

### Experimental

A mixture of phenanthrene-9,10-dione (1.0 g, 4.8 mmol), ammonium acetate (1.48 g, 19.2 mmol), 4-fluorobenzaldehyde (0.62 g, 4.8 mmol) and 3,5-dimethyl aniline (3.82 g, 24 mmol) have been refluxed in ethanol (20 ml) at 353 K. The reaction was monitored by *TLC* and purified by column chromatography using petroleum ether : ethyl acetate (9:1) as the eluent. Yield: 0.78 g (50%). The compound was dissolved in *DMSO* and allowed to slow evaporation and single crystals were grown within a period of one week.

### Refinement

All the hydrogen atoms were geometrically fixed and allowed to ride on their parent atoms with C–H = 0.93-0.96Å with *U*_iso_(H) = 1.5*U*_eq_(C) for methyl H atoms and *U*_iso_(H) = 1.2*U*_eq_(C) for aryl H atoms.

### Crystal data

**Table:**

C_29_H_21_FN_2_	*F*(000) = 872
*M**_r_* = 416.48	*D*_x_ = 1.288 Mg m^−^^3^
Monoclinic, *P*2_1_/*n*	Mo *K*α radiation, λ = 0.71073 Å
Hall symbol: -P 2yn	Cell parameters from 6884 reflections
*a* = 8.5680 (2) Å	θ = 2.6–25.8°
*b* = 10.6070 (3) Å	µ = 0.08 mm^−^^1^
*c* = 23.6900 (6) Å	*T* = 295 K
β = 93.899 (1)°	Block, colourless
*V* = 2147.98 (10) Å^3^	0.30 × 0.20 × 0.20 mm
*Z* = 4	

### Data collection

**Table:**

Bruker Kappa APEXII CCD diffractometer	3777 independent reflections
Radiation source: fine-focus sealed tube	2957 reflections with *I* > 2σ(*I*)
Graphite monochromator	*R*_int_ = 0.034
ω– and φ–scans	θ_max_ = 25.0°, θ_min_ = 2.1°
Absorption correction: multi-scan (*SADABS*; Bruker, 2008)	*h* = −10→10
*T*_min_ = 0.952, *T*_max_ = 0.995	*k* = −12→12
19934 measured reflections	*l* = −28→28

### Refinement

**Table:**

Refinement on *F*^2^	Secondary atom site location: difference Fourier map
Least-squares matrix: full	Hydrogen site location: inferred from neighbouring sites
*R*[*F*^2^ > 2σ(*F*^2^)] = 0.039	H-atom parameters constrained
*wR*(*F*^2^) = 0.109	*w* = 1/[σ^2^(*F*_o_^2^) + (0.047*P*)^2^ + 0.6584*P*] where *P* = (*F*_o_^2^ + 2*F*_c_^2^)/3
*S* = 1.02	(Δ/σ)_max_ = 0.001
3777 reflections	Δρ_max_ = 0.24 e Å^−^^3^
292 parameters	Δρ_min_ = −0.15 e Å^−^^3^
0 restraints	Extinction correction: *SHELXL97* (Sheldrick, 2008), Fc^*^=kFc[1+0.001xFc^2^λ^3^/sin(2θ)]^-1/4^
Primary atom site location: structure-invariant direct methods	Extinction coefficient: 0.0229 (14)

### Special details

**Table:**

Geometry. All s.u.'s (except the s.u. in the dihedral angle between two l.s. planes) are estimated using the full covariance matrix. The cell s.u.'s are taken into account individually in the estimation of s.u.'s in distances, angles and torsion angles; correlations between s.u.'s in cell parameters are only used when they are defined by crystal symmetry. An approximate (isotropic) treatment of cell s.u.'s is used for estimating s.u.'s involving l.s. planes.
Refinement. Refinement of *F*^2^ against ALL reflections. The weighted *R*-factor *wR* and goodness of fit *S* are based on *F*^2^, conventional *R*-factors *R* are based on *F*, with *F* set to zero for negative *F*^2^. The threshold expression of *F*^2^ > σ(*F*^2^) is used only for calculating *R*-factors(gt) *etc*. and is not relevant to the choice of reflections for refinement. *R*-factors based on *F*^2^ are statistically about twice as large as those based on *F*, and *R*-factors based on ALL data will be even larger.

### Fractional atomic coordinates and isotropic or equivalent isotropic displacement parameters (Å^2^)

**Table:**

	*x*	*y*	*z*	*U*_iso_*/*U*_eq_	
F1	−0.12516 (16)	−0.11339 (12)	0.46971 (5)	0.0814 (5)	
N1	−0.17180 (15)	0.00029 (12)	0.20526 (5)	0.0432 (4)	
N2	−0.01474 (14)	0.16068 (12)	0.23196 (5)	0.0397 (4)	
C1	0.13686 (19)	0.43494 (15)	0.31531 (7)	0.0426 (5)	
C2	0.03897 (18)	0.35869 (15)	0.28130 (7)	0.0417 (5)	
C3	0.08624 (17)	0.23859 (14)	0.26818 (6)	0.0384 (5)	
C4	0.22859 (18)	0.19160 (15)	0.28854 (7)	0.0428 (5)	
C5	0.32914 (18)	0.26633 (16)	0.32246 (7)	0.0482 (6)	
C6	0.2809 (2)	0.38706 (16)	0.33513 (7)	0.0486 (6)	
C7	−0.03496 (17)	0.16561 (14)	0.17341 (6)	0.0385 (5)	
C8	0.02988 (18)	0.24785 (15)	0.13278 (7)	0.0424 (5)	
C9	0.1372 (2)	0.34535 (16)	0.14610 (8)	0.0520 (6)	
C10	0.1931 (2)	0.41945 (19)	0.10496 (9)	0.0624 (7)	
C11	0.1435 (3)	0.3995 (2)	0.04904 (9)	0.0740 (8)	
C12	0.0414 (3)	0.3043 (2)	0.03479 (8)	0.0692 (8)	
C13	−0.0186 (2)	0.22404 (17)	0.07527 (7)	0.0504 (6)	
C14	−0.1241 (2)	0.12002 (17)	0.05928 (7)	0.0505 (6)	
C15	−0.1728 (3)	0.0935 (2)	0.00278 (8)	0.0742 (8)	
C16	−0.2694 (3)	−0.0054 (3)	−0.01118 (9)	0.0833 (9)	
C17	−0.3242 (3)	−0.0829 (2)	0.02974 (9)	0.0705 (8)	
C18	−0.2803 (2)	−0.06043 (17)	0.08542 (8)	0.0537 (6)	
C19	−0.18058 (18)	0.04040 (16)	0.10068 (7)	0.0442 (5)	
C20	−0.13220 (17)	0.06682 (14)	0.15854 (6)	0.0395 (5)	
C21	−0.09957 (18)	0.05819 (14)	0.24857 (7)	0.0401 (5)	
C22	−0.10644 (18)	0.01699 (15)	0.30742 (7)	0.0412 (5)	
C23	−0.1144 (2)	−0.11111 (16)	0.31864 (7)	0.0507 (6)	
C24	−0.1214 (2)	−0.15484 (17)	0.37296 (8)	0.0551 (6)	
C25	−0.1211 (2)	−0.07015 (18)	0.41601 (7)	0.0542 (6)	
C26	−0.1164 (3)	0.05626 (19)	0.40719 (8)	0.0627 (7)	
C27	−0.1093 (2)	0.09943 (17)	0.35261 (8)	0.0547 (6)	
C28	0.4870 (2)	0.2170 (2)	0.34406 (10)	0.0739 (8)	
C29	0.0858 (2)	0.56524 (16)	0.33040 (8)	0.0557 (6)	
H2	−0.05829	0.38834	0.26736	0.0501*	
H4	0.25741	0.10985	0.27959	0.0514*	
H6	0.34764	0.43796	0.35778	0.0584*	
H9	0.17076	0.35964	0.18372	0.0624*	
H10	0.26440	0.48327	0.11457	0.0749*	
H11	0.17975	0.45104	0.02102	0.0888*	
H12	0.01026	0.29201	−0.00318	0.0830*	
H15	−0.13831	0.14452	−0.02578	0.0890*	
H16	−0.29900	−0.02076	−0.04906	0.0999*	
H17	−0.39033	−0.14989	0.01960	0.0846*	
H18	−0.31691	−0.11232	0.11328	0.0644*	
H23	−0.11507	−0.16832	0.28888	0.0608*	
H24	−0.12626	−0.24089	0.38017	0.0661*	
H26	−0.11785	0.11237	0.43731	0.0753*	
H27	−0.10636	0.18578	0.34598	0.0656*	
H28A	0.49953	0.22771	0.38435	0.1108*	
H28B	0.49460	0.12914	0.33495	0.1108*	
H28C	0.56759	0.26283	0.32662	0.1108*	
H29A	0.02493	0.56122	0.36291	0.0836*	
H29B	0.17619	0.61712	0.33882	0.0836*	
H29C	0.02357	0.60076	0.29911	0.0836*	

### Atomic displacement parameters (Å^2^)

**Table:**

	*U*^11^	*U*^22^	*U*^33^	*U*^12^	*U*^13^	*U*^23^
F1	0.1151 (10)	0.0821 (9)	0.0484 (7)	−0.0124 (7)	0.0160 (6)	0.0106 (6)
N1	0.0415 (7)	0.0419 (8)	0.0456 (8)	−0.0017 (6)	−0.0016 (6)	−0.0019 (6)
N2	0.0403 (7)	0.0382 (7)	0.0399 (7)	−0.0021 (6)	−0.0023 (6)	−0.0012 (6)
C1	0.0456 (9)	0.0402 (9)	0.0425 (9)	−0.0079 (7)	0.0076 (7)	0.0003 (7)
C2	0.0367 (8)	0.0415 (9)	0.0468 (9)	−0.0007 (7)	0.0021 (7)	0.0017 (7)
C3	0.0359 (8)	0.0394 (9)	0.0396 (8)	−0.0042 (7)	0.0011 (6)	0.0001 (7)
C4	0.0408 (9)	0.0404 (9)	0.0474 (9)	0.0003 (7)	0.0042 (7)	0.0017 (7)
C5	0.0386 (9)	0.0512 (10)	0.0539 (10)	−0.0057 (8)	−0.0027 (8)	0.0066 (8)
C6	0.0463 (9)	0.0503 (10)	0.0486 (10)	−0.0156 (8)	−0.0018 (8)	−0.0014 (8)
C7	0.0358 (8)	0.0382 (9)	0.0412 (9)	0.0061 (7)	−0.0005 (7)	−0.0004 (7)
C8	0.0387 (8)	0.0423 (9)	0.0464 (9)	0.0077 (7)	0.0049 (7)	0.0021 (7)
C9	0.0512 (10)	0.0495 (10)	0.0559 (11)	−0.0006 (8)	0.0079 (8)	0.0031 (8)
C10	0.0623 (12)	0.0551 (12)	0.0715 (14)	−0.0063 (9)	0.0171 (10)	0.0046 (10)
C11	0.0864 (16)	0.0722 (14)	0.0662 (14)	−0.0087 (12)	0.0262 (12)	0.0136 (11)
C12	0.0818 (14)	0.0790 (14)	0.0478 (11)	−0.0010 (12)	0.0121 (10)	0.0069 (10)
C13	0.0497 (10)	0.0559 (11)	0.0463 (10)	0.0089 (8)	0.0084 (8)	0.0036 (8)
C14	0.0500 (10)	0.0582 (11)	0.0430 (9)	0.0104 (8)	0.0017 (8)	−0.0030 (8)
C15	0.0824 (15)	0.0948 (17)	0.0446 (11)	−0.0068 (13)	−0.0011 (10)	−0.0024 (11)
C16	0.0932 (17)	0.1064 (19)	0.0476 (12)	−0.0068 (15)	−0.0140 (11)	−0.0170 (12)
C17	0.0713 (13)	0.0731 (14)	0.0644 (13)	−0.0016 (11)	−0.0145 (11)	−0.0180 (11)
C18	0.0517 (10)	0.0528 (11)	0.0547 (11)	0.0040 (8)	−0.0091 (8)	−0.0088 (8)
C19	0.0400 (9)	0.0462 (9)	0.0456 (9)	0.0095 (7)	−0.0025 (7)	−0.0069 (8)
C20	0.0357 (8)	0.0397 (9)	0.0426 (9)	0.0051 (7)	−0.0012 (7)	−0.0015 (7)
C21	0.0368 (8)	0.0377 (9)	0.0454 (9)	−0.0010 (7)	0.0000 (7)	−0.0011 (7)
C22	0.0364 (8)	0.0428 (9)	0.0443 (9)	−0.0022 (7)	0.0023 (7)	−0.0006 (7)
C23	0.0621 (11)	0.0431 (10)	0.0467 (10)	−0.0068 (8)	0.0022 (8)	−0.0040 (8)
C24	0.0676 (12)	0.0421 (10)	0.0558 (11)	−0.0066 (9)	0.0059 (9)	0.0051 (8)
C25	0.0586 (11)	0.0606 (12)	0.0441 (10)	−0.0065 (9)	0.0093 (8)	0.0051 (9)
C26	0.0854 (14)	0.0546 (12)	0.0503 (11)	−0.0078 (10)	0.0199 (10)	−0.0104 (9)
C27	0.0706 (12)	0.0402 (10)	0.0548 (11)	−0.0014 (9)	0.0160 (9)	−0.0023 (8)
C28	0.0490 (11)	0.0698 (14)	0.0991 (17)	−0.0021 (10)	−0.0219 (11)	0.0089 (12)
C29	0.0626 (12)	0.0446 (10)	0.0609 (11)	−0.0092 (9)	0.0110 (9)	−0.0070 (8)

### Geometric parameters (Å, º)

**Table:**

F1—C25	1.355 (2)	C19—C20	1.433 (2)
N1—C20	1.3745 (19)	C21—C22	1.466 (2)
N1—C21	1.314 (2)	C22—C23	1.387 (2)
N2—C3	1.4376 (19)	C22—C27	1.384 (2)
N2—C7	1.3871 (18)	C23—C24	1.373 (3)
N2—C21	1.380 (2)	C24—C25	1.359 (3)
C1—C2	1.384 (2)	C25—C26	1.358 (3)
C1—C6	1.387 (2)	C26—C27	1.377 (3)
C1—C29	1.500 (2)	C2—H2	0.9300
C2—C3	1.379 (2)	C4—H4	0.9300
C3—C4	1.374 (2)	C6—H6	0.9300
C4—C5	1.386 (2)	C9—H9	0.9300
C5—C6	1.385 (2)	C10—H10	0.9300
C5—C28	1.507 (2)	C11—H11	0.9300
C7—C8	1.438 (2)	C12—H12	0.9300
C7—C20	1.370 (2)	C15—H15	0.9300
C8—C9	1.405 (2)	C16—H16	0.9300
C8—C13	1.420 (2)	C17—H17	0.9300
C9—C10	1.364 (3)	C18—H18	0.9300
C10—C11	1.380 (3)	C23—H23	0.9300
C11—C12	1.363 (3)	C24—H24	0.9300
C12—C13	1.406 (3)	C26—H26	0.9300
C13—C14	1.460 (2)	C27—H27	0.9300
C14—C15	1.403 (3)	C28—H28A	0.9600
C14—C19	1.405 (2)	C28—H28B	0.9600
C15—C16	1.363 (4)	C28—H28C	0.9600
C16—C17	1.378 (3)	C29—H29A	0.9600
C17—C18	1.368 (3)	C29—H29B	0.9600
C18—C19	1.401 (2)	C29—H29C	0.9600
			
C20—N1—C21	105.06 (13)	C23—C24—C25	118.82 (17)
C3—N2—C7	127.18 (12)	F1—C25—C24	118.82 (17)
C3—N2—C21	126.16 (12)	F1—C25—C26	118.79 (16)
C7—N2—C21	106.41 (12)	C24—C25—C26	122.40 (17)
C2—C1—C6	118.25 (15)	C25—C26—C27	118.46 (17)
C2—C1—C29	120.14 (15)	C22—C27—C26	121.37 (17)
C6—C1—C29	121.60 (15)	C1—C2—H2	120.00
C1—C2—C3	119.71 (14)	C3—C2—H2	120.00
N2—C3—C2	119.45 (13)	C3—C4—H4	120.00
N2—C3—C4	119.00 (13)	C5—C4—H4	120.00
C2—C3—C4	121.56 (14)	C1—C6—H6	119.00
C3—C4—C5	119.85 (15)	C5—C6—H6	119.00
C4—C5—C6	118.13 (15)	C8—C9—H9	119.00
C4—C5—C28	120.42 (16)	C10—C9—H9	119.00
C6—C5—C28	121.45 (16)	C9—C10—H10	120.00
C1—C6—C5	122.49 (15)	C11—C10—H10	120.00
N2—C7—C8	131.64 (14)	C10—C11—H11	120.00
N2—C7—C20	105.12 (12)	C12—C11—H11	120.00
C8—C7—C20	123.21 (13)	C11—C12—H12	119.00
C7—C8—C9	124.93 (15)	C13—C12—H12	119.00
C7—C8—C13	115.67 (14)	C14—C15—H15	119.00
C9—C8—C13	119.38 (15)	C16—C15—H15	119.00
C8—C9—C10	121.32 (17)	C15—C16—H16	119.00
C9—C10—C11	119.83 (18)	C17—C16—H16	119.00
C10—C11—C12	120.1 (2)	C16—C17—H17	120.00
C11—C12—C13	122.54 (18)	C18—C17—H17	120.00
C8—C13—C12	116.76 (16)	C17—C18—H18	120.00
C8—C13—C14	121.27 (15)	C19—C18—H18	120.00
C12—C13—C14	121.97 (16)	C22—C23—H23	119.00
C13—C14—C15	122.53 (17)	C24—C23—H23	119.00
C13—C14—C19	120.65 (15)	C23—C24—H24	121.00
C15—C14—C19	116.82 (17)	C25—C24—H24	121.00
C14—C15—C16	121.47 (19)	C25—C26—H26	121.00
C15—C16—C17	121.3 (2)	C27—C26—H26	121.00
C16—C17—C18	119.4 (2)	C22—C27—H27	119.00
C17—C18—C19	120.32 (18)	C26—C27—H27	119.00
C14—C19—C18	120.78 (16)	C5—C28—H28A	109.00
C14—C19—C20	117.35 (15)	C5—C28—H28B	110.00
C18—C19—C20	121.87 (15)	C5—C28—H28C	109.00
N1—C20—C7	111.40 (13)	H28A—C28—H28B	109.00
N1—C20—C19	126.79 (14)	H28A—C28—H28C	109.00
C7—C20—C19	121.81 (14)	H28B—C28—H28C	109.00
N1—C21—N2	112.01 (14)	C1—C29—H29A	109.00
N1—C21—C22	123.78 (14)	C1—C29—H29B	109.00
N2—C21—C22	124.20 (14)	C1—C29—H29C	109.00
C21—C22—C23	118.68 (14)	H29A—C29—H29B	109.00
C21—C22—C27	123.46 (15)	H29A—C29—H29C	109.00
C23—C22—C27	117.85 (16)	H29B—C29—H29C	109.00
C22—C23—C24	121.09 (16)		
			
C20—N1—C21—C22	−178.64 (14)	C7—C8—C13—C14	−1.7 (2)
C20—N1—C21—N2	0.33 (17)	C9—C8—C13—C12	−2.2 (2)
C21—N1—C20—C7	0.17 (17)	C8—C9—C10—C11	0.3 (3)
C21—N1—C20—C19	179.41 (15)	C9—C10—C11—C12	−1.4 (3)
C3—N2—C21—C22	3.7 (2)	C10—C11—C12—C13	0.6 (4)
C7—N2—C21—C22	178.27 (14)	C11—C12—C13—C14	−178.1 (2)
C3—N2—C7—C20	175.21 (13)	C11—C12—C13—C8	1.2 (3)
C21—N2—C7—C20	0.74 (16)	C8—C13—C14—C15	−179.38 (18)
C7—N2—C3—C4	−99.85 (18)	C12—C13—C14—C15	−0.2 (3)
C21—N2—C3—C4	73.6 (2)	C12—C13—C14—C19	179.18 (18)
C21—N2—C7—C8	−176.95 (16)	C8—C13—C14—C19	0.0 (3)
C21—N2—C3—C2	−106.97 (18)	C13—C14—C15—C16	178.9 (2)
C7—N2—C21—N1	−0.70 (17)	C19—C14—C15—C16	−0.5 (3)
C7—N2—C3—C2	79.61 (19)	C13—C14—C19—C18	−179.21 (16)
C3—N2—C21—N1	−175.24 (13)	C13—C14—C19—C20	0.8 (2)
C3—N2—C7—C8	−2.5 (3)	C15—C14—C19—C18	0.2 (3)
C2—C1—C6—C5	−0.5 (3)	C15—C14—C19—C20	−179.75 (17)
C29—C1—C6—C5	178.91 (16)	C14—C15—C16—C17	0.5 (4)
C6—C1—C2—C3	0.1 (2)	C15—C16—C17—C18	−0.3 (4)
C29—C1—C2—C3	−179.29 (15)	C16—C17—C18—C19	0.0 (3)
C1—C2—C3—N2	−178.84 (14)	C17—C18—C19—C20	179.97 (18)
C1—C2—C3—C4	0.6 (2)	C17—C18—C19—C14	0.0 (3)
N2—C3—C4—C5	178.51 (14)	C14—C19—C20—N1	−179.08 (15)
C2—C3—C4—C5	−0.9 (2)	C18—C19—C20—N1	1.0 (3)
C3—C4—C5—C28	−178.70 (16)	C18—C19—C20—C7	−179.86 (15)
C3—C4—C5—C6	0.6 (2)	C14—C19—C20—C7	0.1 (2)
C4—C5—C6—C1	0.1 (3)	N1—C21—C22—C23	34.4 (2)
C28—C5—C6—C1	179.37 (17)	N1—C21—C22—C27	−143.96 (17)
N2—C7—C8—C13	179.98 (16)	N2—C21—C22—C23	−144.46 (16)
C20—C7—C8—C9	−176.01 (16)	N2—C21—C22—C27	37.2 (2)
C20—C7—C8—C13	2.7 (2)	C21—C22—C23—C24	−179.86 (15)
N2—C7—C20—N1	−0.58 (17)	C27—C22—C23—C24	−1.4 (2)
N2—C7—C20—C19	−179.87 (14)	C21—C22—C27—C26	179.74 (18)
C8—C7—C20—N1	177.36 (14)	C23—C22—C27—C26	1.4 (3)
N2—C7—C8—C9	1.3 (3)	C22—C23—C24—C25	0.3 (3)
C8—C7—C20—C19	−1.9 (2)	C23—C24—C25—F1	−178.82 (16)
C9—C8—C13—C14	177.08 (16)	C23—C24—C25—C26	1.0 (3)
C7—C8—C9—C10	−179.91 (17)	F1—C25—C26—C27	178.79 (18)
C13—C8—C9—C10	1.5 (3)	C24—C25—C26—C27	−1.0 (3)
C7—C8—C13—C12	179.11 (17)	C25—C26—C27—C22	−0.2 (3)

### Hydrogen-bond geometry (Å, º)

Cg1 and Cg2 are the centroids of the C7/C8/C13/C14/C19/C20 and C8–C13 rings, respectively.

**Table:**

*D*—H···*A*	*D*—H	H···*A*	*D*···*A*	*D*—H···*A*
C9—H9···*Cg*1	0.9300	2.9700	3.8433	156.00
C6—H6···*Cg*2^i^	0.9300	2.9700	3.6994	155.00

Symmetry code: (i) −*x*+1/2, *y*+1/2, −*z*+1/2.
